# Tract-based analyses of white matter in schizophrenia, bipolar disorder, aging, and dementia using high spatial and directional resolution diffusion imaging: a pilot study

**DOI:** 10.3389/fpsyt.2024.1240502

**Published:** 2024-02-01

**Authors:** Daniel Mamah, ShingShiun Chen, Joshua S. Shimony, Michael P. Harms

**Affiliations:** ^1^ Department of Psychiatry, Washington University School of Medicine, St. Louis, MO, United States; ^2^ Mallinckrodt Institute of Radiology, Washington University School of Medicine, St. Louis, MO, United States

**Keywords:** schizophrenia, bipolar disorder, dementia, diffusion, brain, connectivity, TRACULA

## Abstract

**Introduction:**

Structural brain connectivity abnormalities have been associated with several psychiatric disorders. Schizophrenia (SCZ) is a chronic disabling disorder associated with accelerated aging and increased risk of dementia, though brain findings in the disorder have rarely been directly compared to those that occur with aging.

**Methods:**

We used an automated approach to reconstruct key white matter tracts and assessed tract integrity in five participant groups. We acquired one-hour-long high-directional diffusion MRI data from young control (CON, n =28), bipolar disorder (BPD, n =21), and SCZ (n =22) participants aged 18-30, and healthy elderly (ELD, n =15) and dementia (DEM, n =9) participants. Volume, fractional (FA), radial diffusivity (RD) and axial diffusivity (AD) of seven key white matter tracts (anterior thalamic radiation, ATR; dorsal and ventral cingulum bundle, CBD and CBV; corticospinal tract, CST; and the three superior longitudinal fasciculi: SLF-1, SLF-2 and SLF-3) were analyzed with TRACULA. Group comparisons in tract metrics were performed using multivariate and univariate analyses. Clinical relationships of tract metrics with recent and chronic symptoms were assessed in SCZ and BPD participants.

**Results:**

A MANOVA showed group differences in FA (λ=0.5; p=0.0002) and RD (λ=0.35; p<0.0001) across the seven tracts, but no significant differences in tract AD and volume. Post-hoc analyses indicated lower tract FA and higher RD in ELD and DEM groups compared to CON, BPD and SCZ groups. Lower FA and higher RD in SCZ compared to CON did not meet statistical significance. In SCZ participants, a significant negative correlation was found between chronic psychosis severity and FA in the SLF-1 (r= -0.45; p=0.035), SLF-2 (r= -0.49; p=0.02) and SLF-3 (r= -0.44; p=0.042).

**Discussion:**

Our results indicate impaired white matter tract integrity in elderly populations consistent with myelin damage. Impaired tract integrity in SCZ is most prominent in patients with advanced illness.

## Introduction

Schizophrenia (SCZ) and bipolar disorder (BPD) are both chronic psychiatric illnesses, with some overlapping clinical features. SCZ consists primarily of psychotic symptoms, including delusions, hallucinations, and disorganized behaviors, and often involves cognitive impairment. BPD often involves psychotic features (1), but its core clinical symptoms involve mood abnormalities typically involving periods of mania as well as depression. Many studies have reported genetic overlap between these two disorders (2–4), with associated familial overlap (5). As a result of such findings, increasingly patients with those disorders are studied together.

Numerous studies have investigated white matter integrity in SCZ and BPD. Diffusion imaging studies in SCZ typically report lower fractional anisotropy (FA) in one or more white matter tracts, often regionally, however, the specificity of significant abnormalities has been heterogenous across studies (6, 7). The large, multi-site ENIGMA study involving 4,322 individuals found widespread FA reduction in SCZ, with the anterior corona radiata (*d*=0.40) and corpus callosum (*d*=0.39) showing the greatest effects (8). Abnormal diffusion imaging findings in BPD have been more variable and have shown less robust findings than in SCZ. One meta-analysis reported two clusters of decreased FA, both in the right hemisphere: one close to the parahippocampus and the other close to the anterior cingulate (9). Another voxel-based meta-analytic study reported three significant clusters of decreased FA in BPD: a right posterior temporoparietal cluster and two left cingulate clusters (10). More recently, a mega-analysis across 3,033 individuals collected through the ENIGMA network reported lower FA in 29 regions, most notably within the corpus callosum and cingulum (11). Fewer studies have compared white matter anisotropy across both disorders (12, 13). A recent meta-analysis of diffusion and structural imaging white matter abnormalities in SCZ and BPD found a shared decrease in corpus callosum volume and FA across disorders, as well as SCZ-specific white matter abnormalities involving the left cingulum and the right anterior limb of the internal capsule (ALIC) (13).

Cognitive impairment occurs less commonly in BPD than in SCZ (14), where deficits in a range of domains including attention, working memory, verbal memory, and executive functioning are core features of the disorder and are generally chronologically progressive (15–17). SCZ has also been associated with an increased risk of dementia later in life, including showing precocious onset among younger individuals with the disorder (18–20). SCZ has therefore been hypothesized to be a disorder of accelerated biological aging (18, 21, 22). Accelerated aging is supported by studies reporting shared genetics between SCZ and a variety of age-related diseases (23, 24). Measures of biological aging could prove valuable for assessing SCZ patients’ risk for physical and cognitive decline and for evaluating intervention effectiveness. Direct comparisons of the brains of SCZ patients with those with age-related brain changes using identical imaging methods have however rarely been done.

TRACULA (TRActs Constrained by UnderLying Anatomy) is a software package that uses global probabilistic tractography combined with anatomical priors informed by the individual’s own anatomy to accurately map known fiber tracts in the brain and can evaluate multiple diffusion tensor imaging (DTI) parameter values within these tracts (25). TRACULA has been used in multiple research studies to evaluate different neuropathologies such as temporal lobe epilepsy (26), multiple sclerosis (27), amyotrophic lateral sclerosis (28), and obsessive-compulsive disorder (29). Only one prior study has conducted tract-based analysis in SCZ with TRACULA and used diffusion imaging data collected on the same customized ‘Connectom’ scanner used for the Human Connectome Project’s (HCP) “Young Adult” study. This study found a trend towards lower tract FA in patients, most significantly in the left anterior thalamic radiation, but no significant FA abnormalities in bipolar disorder (12). Two studies have used TRACULA in BPD. One of these found reduced FA particularly in the parietal part of the superior longitudinal fasciculus (SLF) (30a), and most notably in the SLF, cingulum-cingulate gyrus bundles, and corticospinal tracts in another study (31).

Aging has been associated with a generalized reduction in white matter FA and increased radial diffusivity (RD), particularly in later age, attributed primarily to a degeneration of myelin sheaths and loss of myelinated fibers (32–34). In Alzheimer’s dementia, diffusion imaging results have been variable (35), but further decreases in white matter integrity (relative to age-matched controls) are generally reported (36–38). Using TRACULA, reduced tract FA and increased mean diffusivity (MD) has been previously been found with aging non-uniformly across most tracts (39). Furthermore, elderly individuals with Alzheimer’s dementia or mild cognitive impairment had increased MD in the cingulum bundles compared to age-related control subjects (40). While the tract abnormalities with dementia share similarities to that reported for SCZ, these populations have not been directly compared using tract-based methods.

In the current pilot study, we use TRACULA to investigate white matter tract integrity in young healthy controls and individuals with SCZ and BPD, as well as in healthy elderly and a dementia cohort, all collected using the same scanner and acquisition protocol. Based on the most notable abnormalities reported in prior TRACULA studies in psychiatric populations (12, 30, 31), we focus our investigations on seven white matter tracts: the anterior thalamic radiation; three superior longitudinal fasciculi, the dorsal and ventral cingulum bundles, and the corticospinal tract. We hypothesized that previous TRACULA findings in SCZ and BPD will be reproducible in these major fiber tract pathways. Secondly, we hypothesized that white matter abnormalities in aging and dementia will have similar abnormalities as those seen with SCZ, but greater in severity.

## Methods

### Subjects

The study recruited a younger and an older subject cohort, recruited through community advertisements and volunteer databases. The young subject cohort included three groups of 18 to 30-year-old individuals: 28 healthy young control (CON), 22 schizophrenia (SCZ) and 21 bipolar I disorder (BPD). Participants were diagnosed using the Structured Clinical Interview for DSM-IV Axis I Disorders (SCID-IV) (41). To minimize clinical heterogeneity within the BPD group, only participants with a history of euphoric mania (versus mania characterized by primarily irritable mood) were included in the study. Written informed consent was obtained prior to participation, and all study protocols were approved by the Institutional Review Board at the Washington University School of Medicine in St. Louis, MO.

The older cohort (53-84 yrs) included 15 elderly healthy individuals (ELD) and 9 individuals with dementia (DEM). DEM participant diagnoses were ascertained through a review of medical records and included 8 participants with Alzheimer’s disease and 1 participant with Frontotemporal Dementia.

All participants were excluded if they: (a) met DSM-IV criteria for substance dependence or severe/moderate abuse during the prior 3 months; (b) had a clinically unstable or severe general medical disorder; or (c) had a history of head injury with documented neurological sequelae or loss of consciousness.

### Behavioral assessments

Recent symptoms (i.e., in the prior two weeks) were assessed using the Scale for the Assessment of Negative Symptoms (SANS), and the Scale for the Assessment of Positive Symptoms (SAPS) (42). Chronic symptoms (i.e., prior year) were assessed using the Washington Early Recognition Center Affectivity and Psychosis (WERCAP) Screen, both affective (aWERCAP) and psychosis (pWERCAP) components (43–46).

### Image acquisition

Structural T1w MRI images were acquired on a 3T Siemens Prisma with a 32-channel head coil using a 3D MPRAGE sequence (47, 48) (0.8 mm isotropic voxels, TR/TI = 2400/1000 ms, TE = 2.2 ms, flip angle = 8°, FOV = 256 ×240×166 mm, matrix size = 320 ×300, 208 sagittal slices, in-plane (iPAT) acceleration factor of 2). T2w volumes were also acquired at the same spatial resolution using the variable-flip-angle turbo-spin-echo 3D SPACE sequence (49) (TR/TE=3200/564 ms; same FOV, matrix and in-plane acceleration). The dMRI acquisition protocol was substantially similar to our previous study collected on the HCP ‘Connectom’ scanner (12), but with some modifications necessitated by the lower gradient strength of the Prisma scanner (80 mT/m, vs. 100 mT/m for the ‘Connectom’ scanner). The dMRI scans used the multi-band (MB) sequences from the Center for Magnetic Resonance Research, with 1.25 isotropic voxels, TR = 5000 ms, TE = 104 ms, 6/8 partial Fourier, and MB factor = 4. A full dMRI session included 6 runs (each approximately 8.5 min), representing 3 different gradient tables, with each table acquired once with anterior-to-posterior and posterior-to-anterior phase encoding polarities, respectively. Each gradient table includes approximately 90 diffusion weighting directions plus 6 b = 0 acquisitions interspersed throughout each run. Diffusion weighting consisted of 3 shells of b = 1000, 2000, and 3000 s/mm^2^ interspersed with an approximately equal number of acquisitions on each shell within each run. The diffusion directions matched those used in the HCP “Young Adult” and our previous study (12).

### Image preprocessing

The diffusion data were preprocessed using the “DiffusionPreprocessing” stream of the HCPpipelines (v4.3.0) (50, 51), using the QuNex container (v0.91.11). This pipeline includes intensity normalization, susceptibility distortion correction (via FSL’s ‘topup’) (52), and correction for eddy current distortions and motion via FSL’s ‘eddy’ tool (53). We used the advanced ‘eddy’ features of outlier replacement (54), slice-to-volume motion correction (55), and correction for susceptibility-by-movement interactions (56). The b-vectors were rotated to account for motion (57). Finally, the dMRI data was corrected for gradient nonlinearity distortion as part of resampling to the subject’s native T1w space from the HCP structural pipeline output (while maintaining the same 1.25 mm spatial resolution of the dMRI data). Following that preprocessing, processing continued using FSL’s ‘bedpostx’ (58, 59) to estimate the diffusion orientation distribution. Bedpostx was run outside of TRACULA, within the QuNex container (number of fibers per voxel = 3; deconvolution model = 3 (zeppelins); burnin = 3000; rician noise; gradient nonlinearities accounted for).

### TRACULA

**Figure 1:**
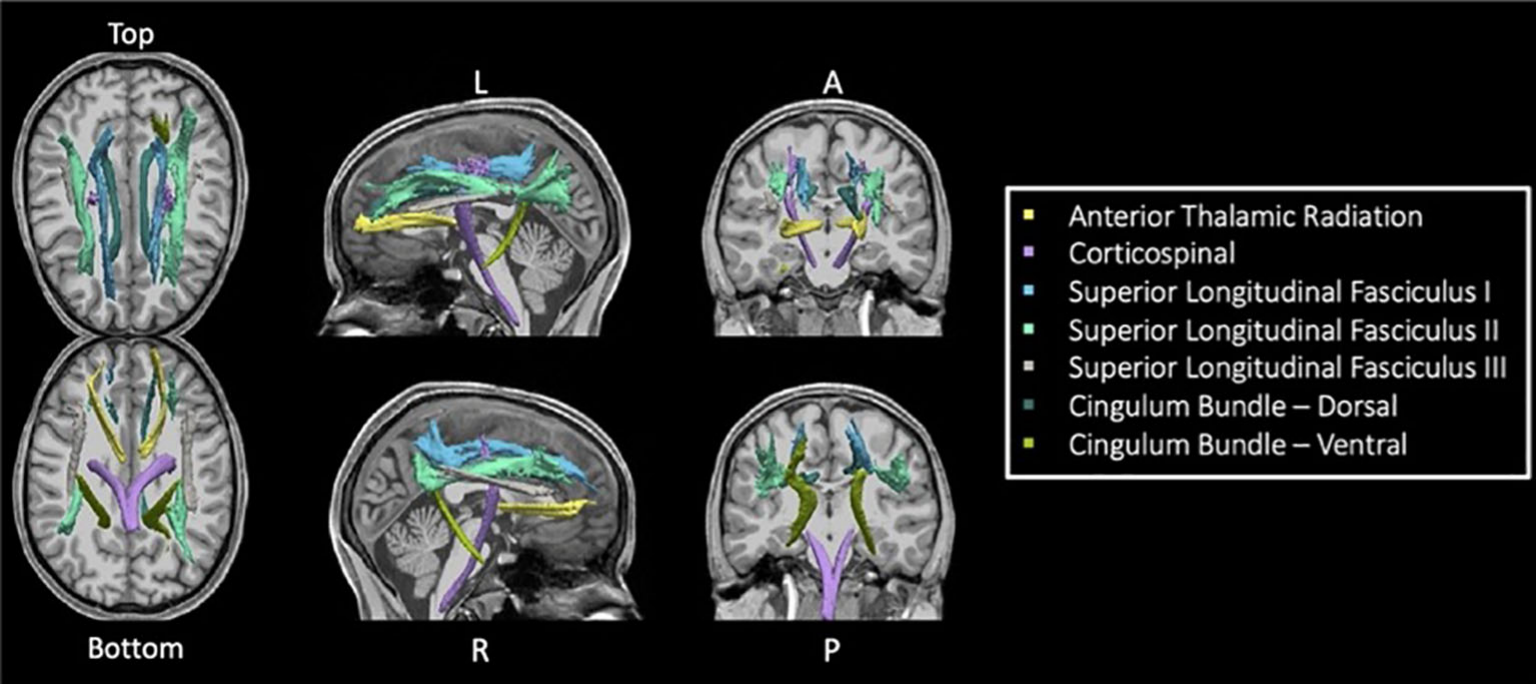
Seven TRACULA tracts assessed. Figure shows the seven reconstructed tracts from TRACULA used in the current analysis.

TRACULA (TRActs Contstrained by UnderLying Anatomy) is an automated method (25) for estimating global probabilistic tractography. This method uses a Bayesian framework for global tractography that determines the connection that best fits two selected endpoints based on the diffusion data. In addition, TRACULA also incorporates prior anatomical knowledge based on manually verified trajectories of tracts in a training set created by Yendiki et al. (25). For every individual, TRACULA reconstructs probabilistic distributions of 18 major white matter tracts. A sample participant’s estimation and identification of white matter tracts is shown in **Figure 1**. More specifically, TRACULA uses the endpoints established in the training set’s tracts, and transforms them into each individual’s native space. Then, TRACULA establishes probabilistic streamlines that are constrained by the relative positions of white-matter pathways to surrounding anatomical structures (obtained from the individual’s own FreeSurfer segmentation) and uses control points to control the allowed curvature of the tract. It does not presume exact tract spatial location or shape, so the trajectory of the tract is only restricted with respect to the surrounding anatomical structures. This allows for variation across individuals while still establishing the same tracts for across-individual comparison.

Given that preprocessing of the diffusion data was implemented using the HCPpipelines (to enable use of more advanced preprocessing features), we only used the TRACULA steps specifically necessary to generate the path distributions (i.e., “prep -prior” to estimate anatomical neighborhood prior for each pathway of interest, and the “path” step to generate the path distributions, using TRACULA from FreeSurfer v6.0). FSL’s “dtifit” was applied to perform least-squares tensor estimations, specifically eigenvectors, eigenvalues and DTI parameters (FA, AD, and RD), using just the b=0 and b=1000 s/mm^2^ shells, since the tensor model is not valid for high b-values. (Note however that all shells were used as input to ‘bedpostx’ and thus contributed to the estimation of the path distributions). FA is a commonly used DTI metric that establishes the directional asymmetry of water diffusion at each voxel (10, 30, 60, 61). Tract volumes, and average values for FA, RD, and AD within the 20% posterior distribution for each path (tract) of interest were computed as the final step of TRACULA (25).

### Statistical analysis

All statistical analyses were done using SAS 9.4 (SAS Institute Inc., Cary, NC). To compare each tract metric (i.e., tract volume, FA, RD, and AD) across groups, multiple analyses of variance was used with the seven tracts as the dependent variables, group as an independent (“class”) variable, and sex as a covariate (i.e., MANCOVA). For tract volume comparisons, intracranial volume was also included as a covariate. *Post-hoc* pairwise analyses were done when the MANCOVA met statistical significance (p<0.05) for the diagnostic group effect, uncontrolled for multiple comparisons. Z-scores were generated using three younger groups only. Relationships between mean FA for each tract and clinical measures (i.e., SAPS – positive and disorganized, SANS, WERCAP-affectivity, and WERCAP-psychosis) and age were investigated using Pearson’s correlations.

## Results

### Demographic and clinical profiles

**Table 1** shows demographic and clinical information across groups. Mean age was similar across the young participant groups, and similar across the two older groups. Sex was relatively evenly balanced across groups, other than in the BPD group which had substantially more females than males.

**Table 1:** Demographics and clinical symptoms.

Characteristic	Control (n=28)	Bipolar (n=21)	Schizophrenia (n=22)	Elderly (n=15)	Dementia (n=9)	F/χ^2^	p
**Age (s.d.)**	25.5 (3.3)	27.0 (3.6)	26.7 (4.1)	68.9 (8.2)	64.6 (9.8)	257.9	<0.0001
**Sex (%)**						6.3	0.2
Female	17 (60.7)	17 (81.0)	10 (47.6)	7 (46.7)	5 (55.6)		
Male	10 (39.3)	11 (19.1)	11 (52.4)	8 (53.3)	4 (44.4)		
Symptom severity (s.d)							
SAPS, positive	0	1.0 (1.6)	3.2 (2.3)	0.1 (0.3)	0	21.5	<0.0001
SAPS, disorganized	0.1 (0.6)	1.2 (2.3)	2.2 (2.2)	0.1 (0.5)	0.7 (1.1)	6.2	0.0002
SANS	0.5 (1.3)	2.9 (4.9)	3.9 (4.9)	0.3 (0.7)	3.8 (3.3)	20.6	<0.0001
WERCAP psychosis	1.2 (2.5)	10.9 (10.5)	32.9 (15.1)	0	0.6 (1.1)	50.2	<0.0001
WERCAP affectivity	8.0 (7.3)	28.9 (7.3)	22.4 (9.8)	2.8 (2.8)	6.3 (6.8)	42.9	<0.0001

### Age relationship with tract metrics

Correlations of age with tract volume and FA were assessed using the individuals from the young participant groups: CON, BPD and SCZ.

A Pearson correlation, partialling out group, did not show a significant age correlation with any tract volume. There were also no significant tract volume relationships with age, when correlations were done separately in each group. Tract FA relationships with age were significant only for the SLF-3 (r= -0.24; p=0.046), after partialling out diagnostic group. There were no significant FA relationships with age when correlations were done separately in each group.

Due to a relatively small number of ELD and DEM participants, age correlations were not done in these groups.

### Intracranial and tract volumes

Intracranial and tract volume least-square means, controlled for sex, across groups, is shown in **Table 2**. Intracranial volumes controlled for sex did not differ across groups (F=2.1, p=0.09).

**Table 2:** Intracranial and tract volume least square means across groups, controlled for gender.

Region	Control (n=28)	Bipolar (n=21)	Schizophrenia (n=22)	Elderly (n=15)	Dementia (n=9)
Intracranial Volume*	1,579	1,587	1,698	1,679	1,659
White matter tract (bilateral)**					
Anterior Thalamic Fasc.	1570	1640	1395	1737	1437
Cingulum Bundle (Dorsal)	1813	1879	1902	1844	1705
Cingulum Bundle (Ventral)	1059	1139	1179	1087	1220
Corticospinal Tract	4041	4367	4882	5266	4944
Superior Long Fasc. I	3180	3271	3161	3099	3114
uperior Long Fasc. II	4934	4534	4853	4966	5012
Superior Long. Fasc. III	1776	1770	1852	1696	1640

*Volumes are given in cm^3^.

**Volumes are given in mm^3^.

A MANCOVA comparing the seven tract volumes across all five participant groups did not meet statistical significance (Wilks’ Lambda = 0.7; p=0.5). MANCOVA results were similar when tract volumes were controlled for intracranial volume (p=0.5).

**Figure 2:**
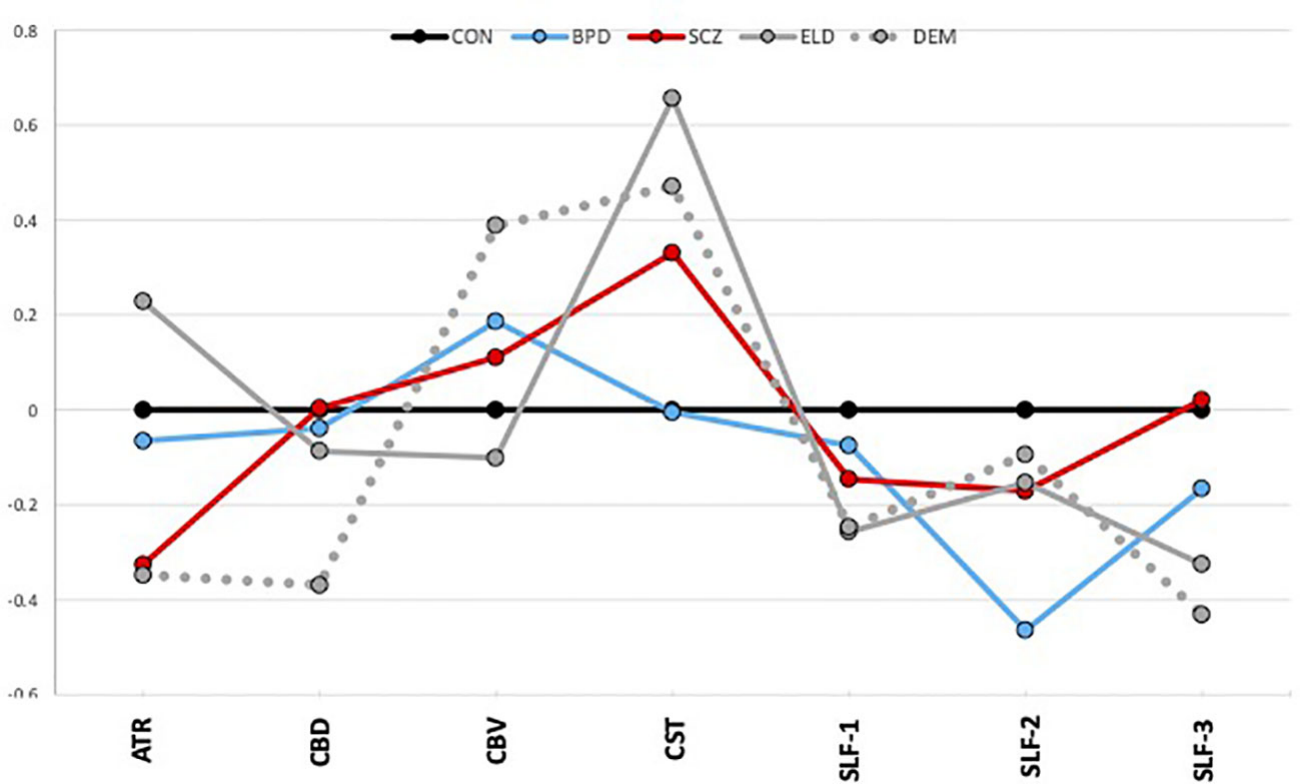
Tract volumes controlled for intracranial volume (z-scores). The graph shows z-scores of tract volumes (normalized to the mean of the healthy young control group). Black=young control; Blue=bipolar disorder; Red=schizophrenia; Gray, solid=elderly control; and Gray, dotted=dementia.

**Figure 2** depicts average group z-scores of tract volumes divided by intracranial volumes to correct for brain size, and adjusted such that CON group z-scores are zero.

### Tract FAs

**Figure 3:**
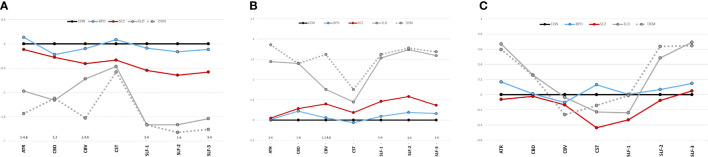
**(A)** Tract diffusion imaging metrics across groups (z-scores). **(A)** Fractional anisotropy. **(B)** Radial diffusivity. **(C)** Axial diffusivity. The graphs show z-scores of each diffusion metric (computed as in **Figure 2**). Black=young control; Blue=bipolar disorder; Red=schizophrenia; Gray, solid=elderly control; and Gray, dotted=dementia. ^1^Significant difference (p<0.05) between CON vs. ELD. ^2^Significant difference (p<0.05) between CON vs. DEM. ^3^Significant difference (p<0.05) between BPD vs. ELD. ^4^Significant difference (p<0.05) between BPD vs. DEM. ^5^Significant difference (p<0.05) between SCZ vs. ELD. ^6^Significant difference (p<0.05) between SCZ vs. DEM.

A MANCOVA of all five groups’ tract FAs showed a significant effect (Wilks’ Lambda = 0.5; p=0.0002). **Figure 3A** depicts group z-scores (relative to CON) for each tract FA. *Post-hoc* pairwise comparisons (Student’s t-tests) showed statistically significant group effects for six tracts as shown in **Table 3** – the ATR, CBD, CBV, SLF-1, SLF-2, and SLF-3. Only values for the CST were non-significant. As depicted in **Figure 3A**, *post hoc* results were driven by significant pairwise FA group differences between ELD or DEM and CON, BPD, and/or SCZ groups.

**Table 3:** Tract diffusion imaging metrics across groups.

Tract	Control (n=28)	Bipolar (n=21)	Schizophrenia (n=22)	Elderly (n=15)	Dementia (n=9)	F	p
Anterior Thalamic Fasc.							
FA	0.487 (0.03)	0.490 (0.03)	0.484 (0.03)	0.461 (0.02)	0.449 (0.04)	6.0	0.0002*
RD^a^	0.495 (0.02)	0.496 (0.02)	0.498 (0.02)	0.540 (0.02)	0.550 (0.02)	21.5	<0.0001*
AD^a^	1.11 (0.05)	1.11 (0.04)	1.10 (0.04)	1.14 (0.05)	1.14 (0.07)	1.8	0.1
Cingulum Bundle (Dorsal)							
FA	0.567 (0.04)	0.560 (0.03)	0.558 (0.03)	0.529 (0.03)	0.530 (0.04)	4.4	0.003*
RD^a^	0.452 (0.03)	0.460 (0.03)	0.462 (0.03)	0.501 (0.03)	0.499 (0.04)	9.2	<0.0001*
AD^a^	1.21 (0.06)	1.21 (0.05)	1.21 (0.05)	1.22 (0.08)	1.22 (0.08)	0.3	0.9
Cingulum Bundle (Ventral)							
FA	0.516 (0.04)	0.513 (0.03)	0.501 (0.04)	0.489 (0.04)	0.459 (0.03)	5.0	0.001*
RD^a^	0.511 (0.03)	0.513 (0.03)	0.523 (0.04)	0.539 (0.030	0.568 (0.03)	7.0	<0.0001*
AD^a^	1.22 (0.07)	1.22 (0.04)	1.21 (0.05)	1.22 (0.07)	1.21 (0.06)	0.1	0.97
Corticospinal Tract							
FA	0.571 (0.04)	0.575 (0.05)	0.557 (0.04)	0.552 (0.04)	0.547 (0.04)	1.4	0.3
RD^a^	0.457 (0.03)	0.454 (0.04)	0.460 (0.03)	0.472 (0.03)	0.05 (0.03)	1.6	0.2
AD^a^	1.25 (0.06)	1.25 (0.06)	1.22 (0.06)	1.23 (0.07)	1.24 (0.07)	1.0	0.4
Superior Long Fasc. I							
FA	0.551 (0.02)	0.549 (0.02)	0.538 (0.03)	0.512 (0.02)	0.512 (0.02)	11.9	<0.0001*
RD^a^	0.476 (0.02)	0.477 (0.02)	0.487 (0.02)	0.518 (0.02)	0.519 (0.02)	15.9	<0.0001*
AD^a^	1.17 (0.04)	1.17 (0.03)	1.15 (0.03)	1.16 (0.04)	1.17 (0.04)	0.5	0.7
Superior Long Fasc. II							
FA	0.489 (0.02)	0.484 (0.02)	0.473 (0.03)	0.447 (0.02)	0.443 (0.02)	12.7	<0.0001*
RD^a^	0.493 (0.02)	0.498 (0.03)	0.511 (0.03)	0.548 (0.02)	0.598 (0.02)	20.4	<0.0001*
AD^a^	1.08 (0.04)	1.08 (0.03)	1.08 (0.03)	1.10 (0.04)	1.10 (0.04)	1.4	0.2
Superior Long. Fasc. III							
FA	0.534 (0.03)	0.531 (0.02)	0.520 (0.02)	0.496 (0.02)	0.491 (0.02)	11.0	<0.0001*
RD^a^	0.500 (0.02)	0.500 (0.02)	0.502 (0.02)	0.542 (0.03)	0.554 (0.02)	17.0	<0.0001*
AD^a^	1.09 (0.04)	1.09 (0.04)	1.09 (0.03)	1.12 (0.05)	1.11 (0.05)	1.8	0.1

Values are given in averages (standard deviations).

FA, fractional anisotropy; RD, radial diffusivity; AD, axial diffusivity.

a 10^-3^mm^2^/s.

*p<0.005 significance with ANOVA.

Significant pairwise group differences were not observed between any of the younger participant groups. The average FA of each tract was lower in the SCZ group compared to the CON group, particularly in the three SLF tracts (see **Figure 3A**), though none of these differences met statistical significance.

### Tract RDs

Results of a MANOVA of all five groups’ tract RDs showed statistical significance (Wilks’ Lambda = 0.35; p<0.0001). **Figure 3B** depicts group z-scores for each tract for RD. *Post-hoc* analyses showed statistically significant group effects for six tracts as shown in **Table 3** – the ATR, CBD, CBV, SLF-1, SLF-2, and the SLF-3. Only CST RD group differences with non-significant. As depicted in **Figure 3B**, results of *post-hoc* analyses were driven by significant pairwise RD group difference between ELD or DEM and CON, BPD and/or SCZ. Significant pairwise effects were not observed between any of the younger participant groups. The average RD of each tract was higher in the SCZ group compared to the CON group, particularly in the three SLF tracts (see **Figure 3B**), though none of these differences met statistical significance.

### Tract ADs

Results of a MANOVA of all five groups’ tract ADs did not show significant group effects (Wilks’ Lambda = 0.74; p=0.5). **Figure 3C** depicts group z-scores for each tract for AD. Mean tract AD in each group is shown in **Table 3**.

### Clinical relationship with tract volume

Pearson’s correlations were done to investigate relationships of tract volume with each of the five symptoms listed in **Table 1**. In SCZ subjects, a significant correlation was only found for ATR volume with SAPS positive symptoms (r=0.47; p=0.028), which remained even after partialling out sex (r=0.48; p=0.03). When both SCZ and BPD subjects were assessed together, no significant clinical correlations were observed.

### Clinical relationship with tract FA

**Figure 4:**
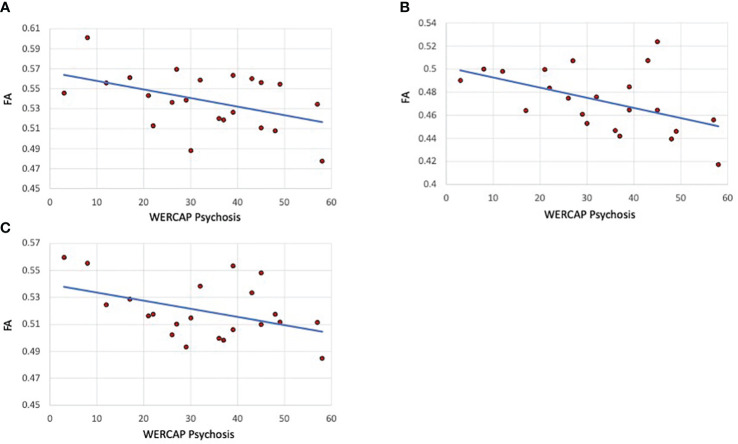
Psychosis correlations with SLF I, II and III fractional anisotropy in schizophrenia patients. **(A)** SLF I **(B)** SLF-II. **(C)** SLF-III. The graphs show scatterplots with regression line between fractional anisotropy within the specific tract and chronic psychosis scores (i.e., over last twelve months) based on the WERCAP screen in schizophrenia participants only.

The correlation of tract FA with the same five symptom measures was also assessed. In SCZ subjects, a significant correlation was only found for WERCAP positive symptoms and FA in the SLF-1 (r= -0.45; p=0.035), SLF-2 (r= -0.49; p=0.02) and SLF-3 (r= -0.44; p=0.042). These relationships are shown in **Figure 4**. When both BPD and SCZ were included in the analyses, the strength of correlations decreased in the SLF-1 (r= -0.33; p=0.029), SLF-2 (r= -0.36; p=0.02) and SLF-3 (r= -0.29; p=0.059).

## Discussion

Our study provides preliminary data comparing diffusion imaging metrics across younger psychiatric populations and older cohorts using an automated tract-based analysis. We found that the volumes of the white matter tracts did not differ significantly across groups, whereas there were significant differences in tract fractional anisotropy across the various tracts studied. This contrasts with several other studies which have reported decreased white matter volumes, though results have been inconsistent and highly variable. SCZ has been associated with a reduction of prefrontal or frontal white matter (62–65), temporal white matter (62), or posterior brain white matter (66) volumes. Others have reported reduced white matter volume in regions corresponding to the inferior longitudinal fasciculus in first-episode SCZ (67) or to the left fronto-occipital fasciculus in prodromal individuals who converted to psychosis (68). Studies of aging and dementia have also been associated with reductions in white matter volume (34, 69–71). Notably, white matter volumes have been reported to gradually increase in the first 40 years of life and then rapidly decrease after 60 years (70–72). The absence of substantial tract volume differences between our younger and older cohorts in our study was therefore unexpected and suggests that tract-wide volume loss may not be observed until after age 70. As our study involved measures of entire tracts, minor white structural changes in disease are likely diluted by the inclusion of less affected parts of the tracts in the analyses, as has been shown by comparing tract-based analyses results to that of voxel-based analyses (12).

Our study found a substantially reduced FA in most white matter tracts in elderly and dementia participants compared to the younger cohorts, with findings in dementia not significantly lower than in the healthy elderly population. Similarly, we found an increase in RD in the older cohorts, indicating an increase in the water diffusion in the direction perpendicular to the direction of the white matter fiber. These findings are consistent with the generalized reduced white matter integrity reported with old age (32, 34), attributed primarily to the degeneration of myelin sheaths and loss of myelinated fibers (33).

We did not find significant diffusion abnormalities in SCZ or BPD participants. FA in SCZ trended towards lower values, and RD towards higher values, suggesting potential myelin degeneration in this population, albeit to a relatively mild degree. Here again, finding statistically significant diffusion metrics in a whole white matter tract would be less likely if there are regions across the tract that are unaffected or only minimally affected. The white matter regions affected in SCZ have been highly variable across studies, and rarely involve universal white matter abnormalities (6, 7). In general, findings have implicated prefrontal and temporal lobes and the fiber tracts connecting these regions. Analysis of data from the ENIGMA schizophrenia DTI work group of 2,359 healthy controls and 1,963 schizophrenia patients from 29 independent international studies, reported that FA reductions in SCZ are widespread, and involved all major WM fasciculi, with the anterior corona radiata and corpus callosum showing the greatest effects (8). An earlier meta-analytic study found significant FA reductions primarily in two regions: the left frontal deep white matter and the left temporal deep white matter in SCZ (6). Similarly, a meta-analytic study of first-episode psychosis reported FA reduction in the right limbic white matter and left temporal white matter (73). Regarding BPD, diffusion imaging abnormalities have generally been found to be less severe than that found in SCZ (9–11), consistent with the results of our current study.

Tract-based white matter assessments in psychiatric disorders, as described in this study, are relatively infrequent. Another study using TRACULA in twenty-four SCZ patients similarly did not find significant tract effects compared to thirty healthy controls. That study however found a trend toward lower FA in the ATR (12), a tract that was the least affected in our current study. This variability is consistent with the hypothesis that the specific white matter regions most affected in schizophrenia tend to vary widely across patients. A failure to achieve statistically significant findings in schizophrenia using TRACULA may thus be related to heterogeneity across individuals combined with the relatively low sample size. Two substantially larger TRACULA studies of bipolar disorder subjects (with sample sizes of 96 and 72) have shown significant findings across several tracts (30, 31), which was not observed in a smaller TRACULA study of thirty-three bipolar disorder subjects (12). This suggests that tract-based diffusion imaging studies require a larger sample size, compared to that required for voxel-based studies, to detect groupwise FA differences (12).

We however found a significant moderate correlation between chronic psychotic symptom severity and FA in each of the three SLF tracts, which was not observed with more recent symptoms. The WERCAP Screen estimates chronic symptoms over the last year and may therefore more accurately capture cumulative psychopathology compared to the SAPS which estimates recent symptoms (i.e. over the last two weeks), which may be more indicative of state-related changes. The SLF is the largest associative fiber bundle system in the brain, and connects the frontal, temporal, and parietal lobes within the same hemisphere (74, 75). The main functions supported by the regions connected by the SLF are visual and spatial cognition, attention processes, control of motor processes and executive functions, and language functions (76). SLF-I represents the dorsal division, connecting the superior parietal and superior frontal lobes, and appears to be involved with regulating motor behavior. The SLF II originates in the caudal-inferior parietal cortex and terminates in the dorsolateral prefrontal cortex and appears to be involved in visuospatial attention. The SLF-III is the most ventral, extending from the supramarginal gyrus, anterior to the angular gyrus, to the ventral premotor and prefrontal areas. Frontoparietal dysconnectivity has been attributed to SCZ etiopathogenesis (76), and impaired SLF has been found in SCZ in several studies (7, 8, 77–79) and associated with psychotic symptom severity (79, 80). Our results are consistent with these findings and suggest that decreased integrity of SLF tracts may indicate a more advanced symptom profile.

Our study and its interpretation have some limitations. Firstly, the sample size used in our study was only modest for each group (n=28 or fewer) and thus may not have had sufficient power to detect significant group differences, particularly since the white matter regions affected are highly variable across individuals. Nevertheless, our study showed strong trends towards lower FA values in schizophrenia, and FA in the SLF showed a moderate inverse correlation with psychotic symptom severity. In the future, larger tract-based studies of diffusion data acquired similarly are needed to identify significant group effects, as well as subtypes of patients with unique patterns of white matter impairment. Secondly, results from tract-based methods may miss white matter damage if it does not involve a substantial portion of the tract since the healthier parts of the tract could dilute the effect. Thus, combining tract-based methods with voxel-based methods would overcome the disadvantages of each method, facilitating the estimation of both general and regional tract integrity (12). Thirdly, our study did not account for potential confounders in our analyses, including substance and medication use which may have influenced the findings. Antipsychotic use, for example, has been reported to increase tract FA (81, 82), while others have reported a subtle loss of white matter integrity (83). Diffusion studies have also found poorer integrity of white matter in cannabis users compared to non-users (84, 85). In addition, potential protective factors for white matter abnormalities including psychotherapy (86, 87) or cognitive training (88) may have been a confounder in our study, and such data were unavailable from our participants for analyses.

In conclusion, using the automated tractography tool TRACULA, our study showed significantly impaired white matter integrity with aging, suggesting demyelination. The pattern of white matter abnormalities in schizophrenia was similar to that in aging, but was much lesser in severity and did not meet statistical significance. In the future, larger sample sizes and aggregation with other data sets are recommended to increase the power to detect group differences.

## Data availability statement

The raw data supporting the conclusions of this article will be made available by the authors, without undue reservation.

## Ethics statement

The studies involving humans were approved by Washington University Institutional Review Board. The studies were conducted in accordance with the local legislation and institutional requirements. The participants provided their written informed consent to participate in this study.

## Author contributions

DM wrote the original draft and conducted most of the statistical analyses. SC conducted neuroimaging processing and analyses. JS guided imaging processing and analyses. MH oversaw neuroimaging acquisition, processing, and analyses. All authors contributed to the article and approved the submitted version.
